# Dental Disease as a Clinical Marker for Coronary Artery Disease Severity: A Narrative Review of Current Evidence and Mechanisms

**DOI:** 10.3390/medicina61091714

**Published:** 2025-09-20

**Authors:** Corina Cinezan, Camelia Bianca Rus, Ioana Tiberia Ilias, Alexandra Cinezan

**Affiliations:** 1 Department of Medical Disciplines, Faculty of Medicine and Pharmacy, University of Oradea, 410073 Oradea, Romania; rus.cameliabianca@student.uoradea.ro (C.B.R.); ioana.ilias@didactic.uoradea.ro (I.T.I.); 2 Clinical County Emergency Hospital Bihor, 410169 Oradea, Romania; 3 Doctoral School of Biological and Biomedical Sciences, University of Oradea, 410087 Oradea, Romania; 4 Faculty of Dental Medicine, University of Medicine and Pharmacy, 400012 Cluj-Napoca, Romania

**Keywords:** tooth loss, coronary artery disease, periodontal disease, systemic inflammation, atherosclerosis, cardiovascular risk, SYNTAX score, Gensini score, oral health, edentulism

## Abstract

*Background and Objectives*: Coronary atherosclerosis remains a leading cause of global morbidity and mortality. Chronic systemic inflammation has emerged as a key factor in atherosclerosis development. Tooth loss—often the final consequence of periodontitis—has been proposed as a potential clinical marker of systemic inflammation and cardiovascular risk. Objective: This narrative review synthesizes the available literature on the relationship between tooth loss and coronary artery disease (CAD) severity, exploring biological mechanisms, key epidemiological findings, and clinical implications. *Materials and Methods*: We reviewed observational studies, meta-analyses, and clinical reports assessing whether tooth loss is predictive of CAD severity and adverse outcomes. *Results*: A consistent association is reported between tooth loss and increased coronary involvement. Proposed mechanisms include periodontal inflammation, dysbiosis, systemic inflammatory responses, and translocation of oral bacteria. However, confounders such as smoking, diabetes, and socioeconomic status complicate causality. *Conclusions*: Tooth loss may serve as a simple, non-invasive clinical indicator of systemic inflammation and CAD severity. Incorporating oral health evaluation into cardiovascular risk assessment could enhance early detection and prevention strategies. Further longitudinal and interventional studies are required to establish causality and inform clinical guidelines.

## 1. Introduction

Coronary artery disease (CAD) remains the leading cause of morbidity and mortality worldwide, accounting for millions of deaths annually despite substantial advances in prevention, diagnosis, and treatment [1,2]. While classical cardiovascular risk factors such as hypertension, diabetes mellitus, smoking, dyslipidemia, obesity, and sedentary lifestyle are well established, they do not fully explain the burden of disease [1,3]. Consequently, there has been growing interest in identifying novel, easily measurable, and cost-effective clinical markers that could refine cardiovascular risk stratification and enable earlier intervention [2].

Over the past two decades, chronic systemic inflammation has emerged as a central driver in the initiation, progression, and destabilization of atherosclerotic plaques [4]. Inflammatory processes influence endothelial function, lipid metabolism, and thrombotic potential, linking diverse chronic conditions to cardiovascular outcomes. Among these, oral diseases—particularly periodontitis—have attracted considerable attention as potential contributors to atherosclerosis and its clinical manifestations [4,5,6].

Periodontitis is a common, progressive inflammatory disease of the tooth-supporting structures, triggered by a dysbiotic subgingival biofilm and sustained by an aberrant host immune response [4,7,8]. This process leads to connective tissue destruction, alveolar bone resorption, and, ultimately, tooth loss if untreated. Epidemiological data indicate that severe periodontitis affects 10–15% of adults globally, with higher prevalence in older populations and in socioeconomically disadvantaged groups [7,9]. Importantly, the impact of periodontitis extends beyond the oral cavity: bacterial by-products, whole microorganisms, and pro-inflammatory mediators such as interleukin-6 (IL-6), tumor necrosis factor-alpha (TNF-α), and C-reactive protein (CRP) can disseminate into systemic circulation, contributing to low-grade inflammation and vascular injury [8,10,11,12].

Tooth loss, often the final and most visible outcome of untreated periodontal disease, serves as a cumulative marker of lifetime oral pathology and, by extension, of chronic inflammatory burden [7,13]. Because it can be easily observed and quantified in a clinical setting without specialized equipment, tooth loss has been proposed as a simple, non-invasive surrogate indicator of systemic health, including cardiovascular risk [8,14]. Several observational studies have demonstrated a significant association between the extent of tooth loss and both the presence and severity of CAD, as quantified by means of angiographic indices such as the SYNTAX and Gensini scores [15,16,17,18].

From a public health perspective, this potential link carries important implications. In low-resource settings where access to advanced cardiovascular diagnostics is limited, oral health evaluation could provide a low-cost adjunctive tool for identifying individuals at heightened cardiovascular risk [19]. Moreover, the hypothesis that oral disease contributes causally to atherosclerosis raises the possibility that targeted periodontal treatment could have beneficial effects on vascular health—an area currently under active investigation.

This narrative review aims to synthesize the existing literature on the relationship between tooth loss and CAD severity, outline plausible biological mechanisms connecting oral pathology to atherogenesis, and discuss the potential role of oral health assessment in cardiovascular risk stratification. By integrating current evidence from epidemiological studies, mechanistic research, and clinical observations, we seek to clarify whether tooth loss can serve as a meaningful clinical marker and highlight priorities for future research.

## 2. Materials and Methods

### 2.1. Search Strategy and Information Sources

We performed a systematic literature search of MEDLINE/PubMed, Embase, Web of Science (or Scopus), and the Cochrane Library to identify observational studies, meta-analyses, and clinical reports examining the association between tooth loss (or edentulism) and coronary artery disease (CAD) severity. The search window extended from 2000 to 2024. Additional sources included manual screening of reference lists from identified articles and previous reviews and searches of conference abstracts when available.

An example PubMed search strategy (adaptable for other databases) was as follows:

“missing teeth” OR “tooth loss” OR edentulism * OR “missing tooth” AND “coronary artery disease” OR “coronary artery disease” OR CAD OR atheroscleros OR “coronary atherosclerosis” AND multivessel OR Gensini OR SYNTAX OR severity OR “angiographic score” AND “1 January 2000”; “31 March 2025”.

Search results were exported into a reference manager (EndNote/Zotero/Mendeley) and duplicates were removed prior to screening.

### 2.2. Study Selection and Screening

Two independent reviewers (initials) screened titles and abstracts for relevance. Full texts were retrieved for all potentially eligible records and assessed independently by the same two reviewers. Disagreements were resolved by discussion and, when necessary, by consulting a third reviewer (initials).

### 2.3. Eligibility Criteria (Inclusion/Exclusion)

Inclusion criteria

- Population: adults (≥18 years) assessed for coronary artery disease by means of angiography, CT coronary angiography, or clinically adjudicated CAD.
- Exposure/index test: tooth loss (number of missing teeth, edentulism, partial edentulism) or validated measures of oral health that report tooth count. Studies that used periodontitis as an exposure but also reported tooth loss were eligible.
- Comparator: patients with fewer/no missing teeth or analyses treating tooth loss as continuous or categorical exposure.
- Outcomes: measures of CAD severity (angiographic scores such as SYNTAX, Gensini, number of vessels involved, and extent of stenosis), major adverse cardiovascular events (MACE) stratified by CAD severity, or other clinically meaningful CAD severity indices.
- Study design: observational cohort (prospective or retrospective), case–control, cross-sectional studies, and meta-analyses. Clinical reports and mechanistic studies were included for contextual discussion but excluded from pooled quantitative analysis.
- Timeframe: published between 2000 and 2025, English language (or language with accessible translation).

Exclusion criteria

- Case reports, small case series (<10 participants), editorials, narrative reviews (except for background), in vitro or animal studies (except in mechanistic discussion).
- Studies that only assessed periodontal indices without reporting tooth loss (unless tooth loss data were available upon request).
- Studies in pediatric populations.
- Studies lacking quantitative outcomes related to CAD severity or not reporting extractable data.

We found 8 studies that fit the eligibility criteria.

Syntax score is today a useful angiographic parameter that helps in the proper selection of percutaneous coronary intervention vs. coronary artery bypass grafting according to the severity of coronary artery disease [18].

Gensini score provides data about the severity of coronary artery disease that reflects the degree of extension of atherosclerosis [19].

## 3. Results

### 3.1. Oral Health and Systemic Inflammation

Oral health was previously believed to be separate from systemic health, but mounting evidence now disagrees with this long-standing view [8,11]. Periodontal disease is one of the most severe chronic diseases, affecting oral health and having major public health consequences because it can lead to tooth loss and promote systemic inflammation [7]. This disease affects a high proportion of adults globally and is a leading cause of tooth loss [7,9]. Due to chronic bacterial colonization, periodontal disease induces local inflammation that can be extended systemically by the production of pro-inflammatory mediators such as interleukin-1 (IL-1), interleukin-6 (IL-6), interleukin-8 (IL-8), interleukin-17 (IL-17), leukotrienes, prostaglandins, tumor necrosis factor-alpha (TNF-α), C-reactive protein (CRP), fibrinogen, matrix metalloproteinases (MMPs), Type 1 T helper cells (Th1), or T helper 17 cells (Th17—its cellular response may be a critical factor in inflammatory diseases of the oral mucosa) [11,12].

### 3.2. Mechanisms Linking Periodontal Disease to CAD

**Figure 1:**
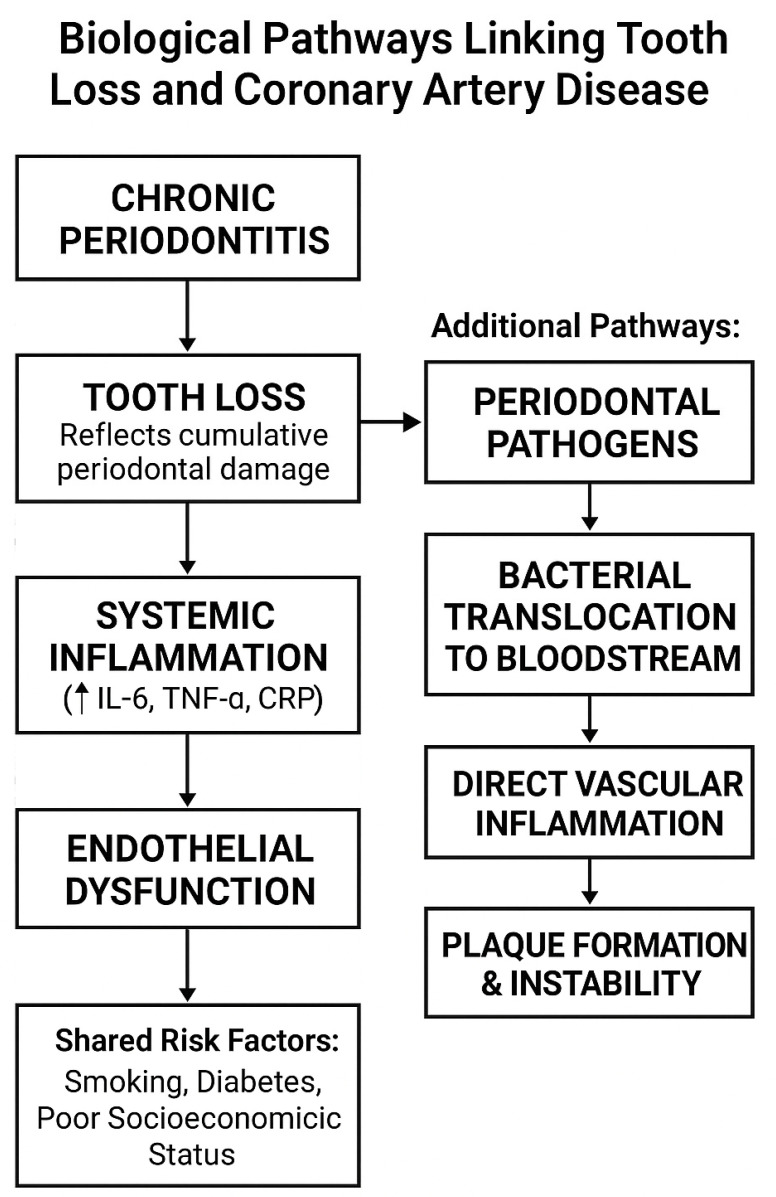
Proposed mechanisms linking periodontal disease and tooth loss to coronary artery disease (CAD). Periodontal inflammation and oral dysbiosis promote systemic dissemination of bacteria (*Porphyromonas gingivalis*) and pro-inflammatory mediators (IL-6, TNF-α, and CRP), leading to endothelial dysfunction, lipid oxidation, immune cross-reactivity, platelet activation, and a prothrombotic state. These processes accelerate atherosclerotic plaque formation, progression, and instability, contributing to higher CAD severity (e.g., SYNTAX and Gensini scores).

Periodontal disease, first considered associated with cardiovascular disease, is now considered a risk factor for atherosclerosis [2,6,20,21]. Pro-inflammatory mediators (IL6, TNF-α, and CRP) driven by periodontal disease are also known promoters of endothelial dysfunction, atherosclerosis, and destabilization of atherosclerotic plaque [4,9,12,22,23]. Endothelial dysfunction is a critical early atherogenic process and appears through blunting the availability of nitric oxide and promoting the expression of adhesion molecules, with consequent monocyte influx into the vessel wall [19,24]. In addition to inflammatory processes, periodontal bacteria such as Porphyromonas gingivalis have been found in atherosclerotic plaques, suggesting the potential of bacterial translocation from the gingival tissue into the blood [11,25,26,27]. These bacteria promote atherosclerosis through oxidation of low-density and high-density lipoproteins [28,29]. Molecular analysis revealed the presence of Porphyromonas gingivalis’ DNA in coronary atherosclerotic plaques [28,30]. This microbial burden may increase vascular inflammation and permeability, instability of the plaque, thrombogenesis, and calcification, all of which play key roles in CAD pathogenesis [31,32,33,34,35,36]. Furthermore, immune cross-reactivity (molecular mimicry) between host proteins and bacterial antigens may play a role in autoimmune-mediated vascular injury [16,37]. Periodontal inflammation modulates platelet activation and creates a pro-thrombotic state, increasing the risk of acute coronary syndromes [6,36]. It also influences lipid metabolism and leads to dyslipidemia, which increases plaque formation [10] Figure 1.

Furthermore, periodontitis and cardiovascular disease share several common risk factors like age, smoking, diabetes mellitus, obesity, and socioeconomic status [8].

These common risk profiles preclude the identification of an immediate causal relationship but also indicate synergistic effects on disease progression [38,39].

Thus, tooth loss—typically resulting from chronic, untreated periodontal disease—may itself serve as an accessible marker for long-standing systemic inflammation and cumulative disease burden, as well as a marker of cardiovascular morbidity and mortality [14,16]. Genetic polymorphisms with an impact upon inflammatory response may place people at risk for both periodontitis and CAD [5].

Understanding the interface between cardiovascular disease and oral health is critical for early risk detection and prevention [8]. Direct causality continues to be investigated, but the biological plausibility of an oral–systemic correlation is confirmed by mechanistic studies and epidemiologic evidence [19,21].

### 3.3. Evidence Linking Tooth Loss Disease to CAD

Tooth loss, often the final result of chronic periodontitis, has also been studied further as an overt and quantifiable marker of systemic health—in particular, cardiovascular disease. While the relationship between periodontitis and CAD has been established, new evidence suggests that the severity of tooth loss may be linked with the extent of coronary disease, such as multivessel disease and higher angiographic scores [3,40,41].

Epidemiological studies investigating the relationship between tooth loss and coronary artery disease (CAD) have employed various designs, like cross-sectional, case–control, and cohort studies. Methodology apart, there is general consistency observed wherein greater tooth loss is associated with greater prevalence and severity of CAD [41,42,43].

Several studies have looked at the relationship between tooth loss and severity of CAD (Table 1).

- Buhlin et al. (2003): Patients undergoing coronary angiography had more severe periodontal disease with deeper periodontal pockets and reduced numbers of teeth compared to controls. The results supported an association between CAD and oral pathology. A limitation of the study could be its size [42].
- Elter et al. (2003): In this cross-sectional study, individuals who were edentulous for more than nine teeth were at higher risk of multivessel CAD and stroke/transient ischemic attacks [44].
- Ylöstalo et al. (2006) established a dose-dependent relationship between missing teeth count and elevated CRP levels, suggesting a systemic inflammatory link [39].
- Gul et al. (2012): A cross-sectional study that reviewed the coronary angiograms of patients and reported that patients with fewer than 10 teeth had considerably higher Gensini scores, compared to those with normal dentition. The limitations of the study are the lack of certain clinical data: door-to-balloon time (important in STEMI cases) or the exact onset of infarction, which can impact the accuracy of the Gensini score [45].
- Liljestrand et al. (2015) followed over 1500 Finnish adults for more than a decade.
- This prospective cohort study demonstrated that edentulous individuals had increased incidence of myocardial infarction, coronary artery calcification, and all-cause mortality, independent of traditional cardiovascular risk factors. Its longitudinal design strengthens the temporal association between tooth loss and cardiovascular outcomes. The most important limitation is the lack of reasons or diagnosis for tooth extractions [15].
- Elevated coronary artery calcium scores were associated with tooth loss by Donders et al. (2020). The study is limited by its size [40].
- Gao et al. (2021) showed an association between coronary heart disease, number of teeth, and periodontitis. The limitations of the study include the fact that the number of teeth does not totally represent oral inflammation; also, the grouping of individuals based on the number of teeth was performed differently in the various articles, and this study ignored the differences in individual teeth [5].
- Shen M et al. (2023) showed that the number of missing teeth is associated with the degree of coronary atherosclerosis, especially in young patients and short-duration diabetic patients. Limitations of the study are the size of the study and the use of Coronary Artery Calcium score as the primary outcome measure; a causal relationship could not be identified due to a lack of information on the time sequence of events [43].

Taken together, these studies suggest that missing teeth would serve as a clinical surrogate marker for the severity of CAD, with patients with high levels of tooth loss being at increased risk for more complex or severe coronary lesions. Importantly, this association remains significant even after controlling for important confounders such as age, smoking, and diabetes.

However, causality cannot be conclusively determined because most existing studies have cross-sectional designs. Prospective studies and randomized trials are required to ascertain whether enhancing oral health—or the prevention of tooth loss—will decrease the extent or severity of coronary artery disease.

Meta-analyses pooling data from multiple studies have begun to give a numerical estimate of the risk increase; individuals with extreme tooth loss may have a 1.5- to 2-fold increase in CAD events compared to those with minimal or no tooth loss [5,13].

### 3.4. Clinical Implications

Tooth loss has important clinical and public health implications [7,46].

Loss of teeth, most often a consequence of advanced periodontal disease or dental caries, has now widely come to be considered not just a sign of compromised oral status but also a clinical marker of systemic conditions like atherosclerosis and its more dangerous effect, coronary artery disease [3]. Periodontal disease is associated with and can be considered a marker of CAD, carotid atherosclerotic disease, and peripheral vascular disease [3,47,48].

The association between cardiovascular disease and oral health results primarily from the periodontal inflammation and infection, which can potentially cause atherosclerosis through systemic dissemination of pro-inflammatory mediators and periodontal pathogens [6,49].

Tooth loss is a simple, non-invasive, and easily observable marker that could potentially augment recognized cardiovascular risk factors. Incorporating oral health examination into standard cardiovascular evaluation could improve identification of high-risk individuals [50].

Various epidemiological studies have shown that individuals with fewer teeth or complete edentulism are at significantly higher risk for CAD.

Bahekar et al. (2007), in a meta-analysis, concluded that periodontitis and tooth loss were associated with a moderately elevated risk of coronary heart disease [49].

Similarly, Holmlund et al. (2010) demonstrated that the remaining number of teeth was inversely correlated with myocardial infarction prevalence even after adjustment for confounding variables such as smoking and diabetes [51].

Several studies—Bengtsson et al. (2021) [49], Romandini et al. (2021) [46], and Yu et al. (2021) [52]—have consistently shown that periodontitis and its surrogate marker, tooth loss, have been found to be associated with cardiovascular mortality and morbidity, and even with all-cause mortality [52].

Clinically, severe tooth loss ought to trigger professionals to evaluate for a broader cardiovascular risk assessment. Because it is a readily visible and non-invasive factor, tooth loss could be a convenient and simple marker, especially in primary care and community health practice [14].

Additionally, the inclusion of dental evaluation in cardiovascular risk stratification plans can enhance earlier detection of patients at risk. In an acute setting, by accurately knowing the patient’s risk, additional human and material resources can be mobilized for their care.

Finally, coordination across disciplines between medical and dental professionals is needed. Dentists may make contributions to cardiovascular risk detection at an early stage, while physicians can highlight the importance of oral health as part of a comprehensive disease prevention program. Cardiologists and dentists should come together in support of patients’ holistic care. Dental clinicians can play a leading role in the early identification of patients at cardiovascular risk and in organizing referrals to medical assessment [53].

Further research is required in establishing standardized procedures for applying oral health indicators in cardiovascular screening.

On the other hand, tooth loss may be a sign of a suboptimal diet, with consequences for cardiovascular health (e.g., low fiber and antioxidant diets) [54].

- It may also be a sign of low socioeconomic status, often with worse outcomes and fewer opportunities to access services [54].
- Psychological impacts of tooth loss (e.g., social withdrawal and depression) are aggravated by cardiometabolic risk profiles [55].

Depression and low self-worth, possibly the mental outcomes of tooth loss, may be the source of unfavorable behavior that may increase cardiometabolic risk [55].

Caution is advised—although tooth loss is visually obvious and non-invasive, its clinical utility as a CAD predictor is unproven. Suggesting it be incorporated into cardiovascular screening tools may be premature without stronger causal evidence. Current guidelines emphasize modifiable risk factors (lipids, blood pressure, smoking, etc.), which offer clearer interventional leverage.

Multidisciplinary collaboration is laudable, but the clinical risk of overdiagnosis or misattribution must be weighed if tooth loss is treated as a proxy for cardiovascular disease without adequate context.

### 3.5. Prevention Approaches

Oral hygiene, periodontal disease management, and prevention of tooth loss could lower cardiovascular morbidity and mortality by lowering systemic inflammatory burden and the initiation, evolution, and complications of atherosclerosis [56].

### 3.6. Core Insights for Cardiologists and General Practitioners

- Tooth loss is more than a dental concern—it can be a visible, easily assessed marker of systemic inflammation and an independent indicator of coronary artery disease (CAD) severity.
- Shared risk factors (age, smoking, diabetes, obesity, and low socioeconomic status) complicate causality, but the biological plausibility is supported by mechanistic evidence involving Th1/Th17 activation, endothelial dysfunction, and pro-thrombotic states.
- Risk stratification: Patients with significant tooth loss, especially when unexplained by trauma or localized pathology, should be considered for enhanced cardiovascular evaluation.
- Multidisciplinary care: Collaboration between dental and medical professionals can improve early detection, risk modification, and patient outcomes.
- Prevention pays: Maintaining periodontal health through regular dental care may reduce systemic inflammatory burden and potentially slow atherosclerotic disease progression.

Bottom line:

For both cardiologists and primary care providers, the oral cavity is a readily accessible “window” into vascular health—missing teeth should prompt looking beyond the gums to the heart.

## 4. Conclusion, Confounding Variables, Limitations, and Future Research

The proposed biological mechanisms—systemic inflammation, endothelial dysfunction, bacterial translocation, and immune mimicry—are supported by preclinical data and small human studies. Yet, no study has definitively shown that periodontal pathogens cause atherosclerosis in vivo. The presence of bacterial DNA in plaques may reflect systemic bacteremia rather than causative infiltration. Moreover, systemic inflammation in periodontitis is not specific to P. gingivalis and could be secondary to other common comorbidities.

Many cited studies [15,42,45] demonstrate statistical associations between tooth loss and angiographic CAD severity or cardiovascular outcomes. However, the following must be noted:

- Most are cross-sectional or retrospective cohort studies, vulnerable to reverse causation and selection bias.
- Key confounders (e.g., smoking, diabetes, and access to dental care) are inconsistently controlled.
- The outcome measures vary widely—from CRP levels to coronary calcium scores to clinical endpoints—making synthesis challenging.

For example, while the [45] study showed a higher Gensini score in patients with fewer than 10 teeth, the study’s cross-sectional design precludes any temporal inference. Similarly, meta-analyses [5] suggest a moderate risk increase, but heterogeneity and publication bias limit interpretability.

Several studies in Table 1 are observational with small samples or unclear methodology. Some used self-reported dental status without validation. Longitudinal studies like Liljestrand et al. are stronger but still limited by residual confounding and lack of intervention.

Limitations of the existing literature include the following: over-reliance on observational data with limited ability to determine temporality; lack of standardization in defining tooth loss severity and CAD measurement; insufficient exploration of effect modification, e.g., by age, sex, or ethnicity; and the fact that few studies distinguish between edentulism from caries vs. periodontitis, despite distinct pathophysiologies.

Recommendations for future research may be as follows:

1. Prospective studies incorporating oral microbiome sequencing and inflammatory biomarkers could strengthen biological plausibility.
2. Randomized trials of periodontal interventions with cardiovascular outcomes are needed to explore causality.
3. Socioeconomic and dietary factors must be integrated into multivariate models to assess the independent effects of oral health.

In conclusion, while tooth loss may reflect poor systemic health, its independent association with CAD severity is not conclusively established. The field requires cautious interpretation of existing data, with an emphasis on eliminating confounding and prioritizing mechanistic and interventional research.

## Figures and Tables

**Table 1:** Relevant studies linking tooth loss and coronary artery disease (CAD—coronary artery disease; TIA—Transient Ischemic Attack; CRP—C-reactive protein).

Author	Year	Study Type	N/Population	Method	Outcome
Buhlin et al. [42]	2003	Observational	96 patients	Coronary Angiography	Fewer teeth, severe periodontal disease
Elter et al. [44]	2003	Cross-sectional	10,906 patients	Multivessel CAD + Stroke/TIA	Increased risk of multivessel CAD
Ylöstalo et al. [39]	2006	Observational	8690 patients	CRP	Dose–response relationship between missing teeth and inflammation
Gul et al. [45]	2012	Cross-sectional	321 patients	Gensini score	Significantly higher Gensini score
Liljestrand et al. [15]	2015	Longitudinal cohort	1500 adults	Cardiovascular Events	All cardiovascular markers increase with edentulism
Donders et al. [40]	2020	Observational	212 adults	Cardiovascular Events	Elevated scores in those with missing teeth
Gao et al. [5]	2021	Meta-analysis	Over 200,000 participants	Periodontitis and the number of teeth alongside CAD	Increased risk of CAD with missing teeth
Shen M et al. [43]	2023	Observational	272 patients	Coronary artery calcium computed tomography scan	More missing teeth → more severe CAD

## Data Availability

The raw data supporting the conclusions of this article will be made available by the authors upon request.
